#  Novel Clinical Monitoring Approaches for Reemergence of Diphtheria Myocarditis, Vietnam

**DOI:** 10.3201/eid2802.210555

**Published:** 2022-02

**Authors:** Ho Quang Chanh, Huynh Trung Trieu, Huynh Ngoc Thien Vuong, Tran Kim Hung, Tu Qui Phan, James Campbell, Caitlin Pley, Sophie Yacoub

**Affiliations:** Hospital for Tropical Diseases, Ho Chi Minh City, Vietnam (H.Q. Chanh, H.T. Trieu, H.N.T. Vuong, T.K. Hung, T.Q. Phan);; Oxford University Clinical Research Unit, Ho Chi Minh City (H.Q. Chanh, H.T. Trieu, J. Campbell, S. Yacoub);; University of Cambridge, School of Clinical Medicine, Cambridge, United Kingdom (C. Pley);; University of Oxford Centre for Tropical Medicine and Global Health, Oxford, United Kingdom (S. Yacoub)

**Keywords:** diphtheria myocarditis, diphtheria, infectious diseases, vaccine-preventable diseases, hemodynamic monitoring, Vietnam, bacteria, Corynebacterium diphtheriae, toxins

## Abstract

Diphtheria is a life-threatening, vaccine-preventable disease caused by toxigenic *Corynebacterium* bacterial species that continues to cause substantial disease and death worldwide, particularly in vulnerable populations. Further outbreaks of vaccine-preventable diseases are forecast because of health service disruptions caused by the coronavirus disease pandemic. Diphtheria causes a spectrum of clinical disease, ranging from cutaneous forms to severe respiratory infections with systemic complications, including cardiac and neurologic. In this synopsis, we describe a case of oropharyngeal diphtheria in a 7-year-old boy in Vietnam who experienced severe myocarditis complications. We also review the cardiac complications of diphtheria and discuss how noninvasive bedside imaging technologies to monitor myocardial function and hemodynamic parameters can help improve the management of this neglected infectious disease.

Diphtheria was once a leading cause of childhood death globally, but cases worldwide have been dramatically reduced over the few past decades by mass vaccination campaigns followed by routine childhood vaccination (*1*). More than 22,000 cases of diphtheria were reported globally in 2019, compared with >97,000 cases in 1980, although both figures are likely to be underestimates (*1*–*3*). However, progress has been grossly uneven; although high-income countries rarely see cases, low- and middle-income countries (LMICs), where the disease remains endemic, frequently grapple with outbreaks (*2*). Annual reported cases have been rising in the past decade, increasing by nearly 5 times during 2010–2019 (Figure 1), likely as a result of improved surveillance and reporting systems, sporadic conflict-associated outbreaks, and other global phenomena such as vaccine hesitancy and migration. Diphtheria is particularly likely to reemerge in settings of conflict or political turmoil, as a result of crowding, inconsistent vaccination, and a lack of public health infrastructure to treat cases and stem further spread (*2*). In recent years, several major outbreaks have occurred in fragile settings, including in Haiti, Venezuela, and Yemen and among Rohingya refugees (*4*–*7*). Vaccination coverage in children has stagnated at ≈86% since 2010 (*2*), and pockets of incomplete vaccination are present in all countries (*3*). The effects of coronavirus disease (COVID-19) on vaccination, case management, and surveillance data quality are not yet known but are likely to reduce vaccination rates even further.

**Figure 1:**
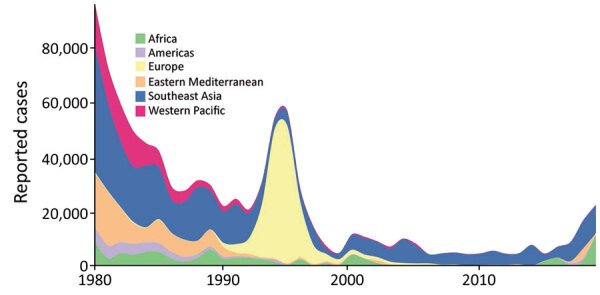
Global and regional epidemiologic trends in reported cases of diphtheria, 1980–2019. Cases shown are those reported to the World Health Organization and the United Nations Children’s Fund.

In Vietnam, during 2004–2019, annual cases of diphtheria ranged from 6 to 53 cases and showed a clear increasing trend (*8*). In the first 9 months of 2020, a large outbreak of 198 cases was reported, mostly in the central highlands (*8*). Although the estimated vaccination rate for 3 doses of the combined diphtheria, tetanus toxoid, and pertussis vaccine was ≈96% in 2014, maintaining vaccination coverage in mountainous and remote areas remains a challenge (*9*). The national public health program has been further disrupted by the COVID-19 pandemic. Several vaccination campaigns have been delayed or cancelled because of restrictions and ongoing public fear of COVID-19.

A diagnosis of diphtheria is made on the basis of clinical features, pathogen isolation, and presence of diphtheria toxin or of the *tox* gene (*10*). Severe diphtheria is usually associated with cardiac and neurologic complications because of the high affinity of diphtheria toxin with theses tissues (*11*). Antitoxin is considered the cornerstone of the prevention of severe complications and death and should be readily available. However, a global shortage of diphtheria antitoxin (DAT) is ongoing, which hinders availability in low-resource settings (*12*). In Vietnam, only a few tertiary hospitals have DAT readily available for early treatment. In recent years, the advance in noninvasive bedside monitoring has enabled early detection of deterioration and more timely intervention, which could improve patient outcomes.

In this article, we describe a case referred to our department at the Hospital for Tropical Diseases in Ho Chi Minh City, Vietnam. After late manifestation of severe diphtheria, the case-patient experienced toxin-mediated complications, including diphtheria myocarditis and neuropathy. We review and discuss the cardiac complications of diphtheria, with a particular focus on how noninvasive bedside imaging technologies to monitor myocardial function and hemodynamic parameters can help improve the management of this neglected infectious disease. We also provide results of a literature search for diphtheria myocarditis based on our search strategy and selection criteria (Appendix).

## The Case-Patient

**Figure 2:**
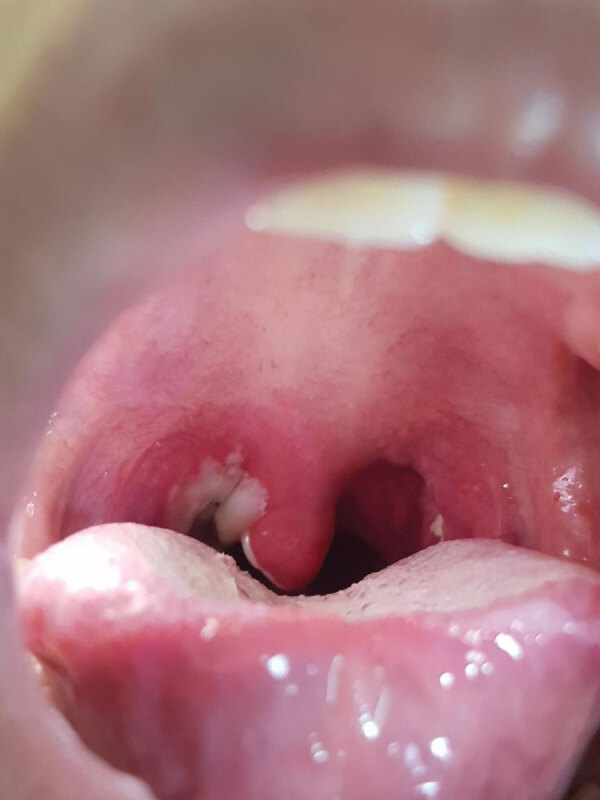
The thick pseudomembrane covering the oropharynx of a 7-year-old boy at admission to a local hospital before his diphtheria myocarditis was diagnosed, Vietnam, 2020.

In July 2020, a 7-year-old boy was brought a provincial hospital in Kon Tum, a highland area of Vietnam, for a 2-day history of a high fever, poor appetite, sore throat, and a progressively swollen neck. He had no unwell contacts, and the boy’s vaccination status was unknown. Clinical examination revealed a swollen neck and pharyngitis with a thick gray pseudomembrane covering the pharynx and tonsils (Figure 2). The patient received a diagnosis of suspected pharyngeal diphtheria and was treated with oral erythromycin (50 mg/kg/d) and prednisolone (5 mg/d). DAT was unavailable. A throat culture yielded *Corynebacterium diphtheriae*. An antibiogram was not available. The Elek test, used to detect the presence of toxigenic *C. diphtheriae* strains, was positive. By day 7 of illness, the patient was afebrile and the pseudomembrane had resolved. His condition deteriorated on day 9 with the onset of chest pain. Cardiac enzymes showed a raised creatinine kinase myocardial band of 60 U/L (reference range 5–25 U/L) and a troponin T level of 3,225.9 pg/mL (reference range <14 pg/mL). An echocardiogram showed normal left ventricular (LV) function and an ejection fraction (EF) of 65.9%. Electrocardiography (ECG) was not performed.

**Figure 3:**
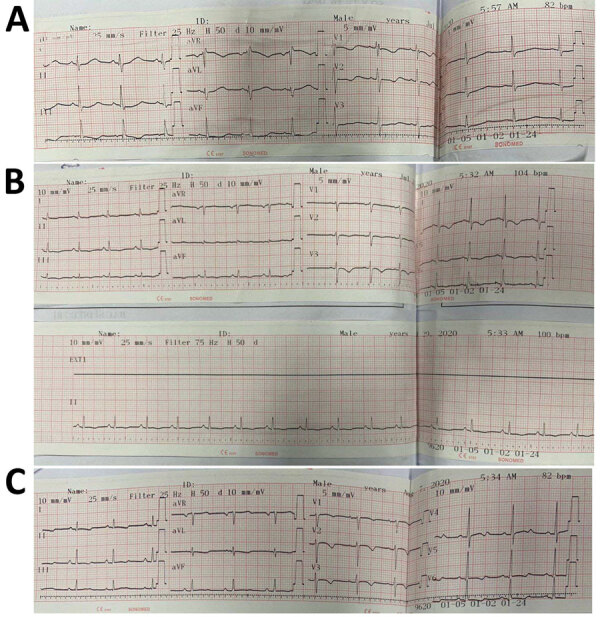
Results of 12-lead electrocardiograms conducted on a 7-year-old boy later diagnosed with diphtheria myocarditis, Vietnam, 2020. A) At hospital admission (day 10 of illness), electrocardiography showed an incomplete right bundle branch block (RSR) in V1–3 with QRS duration of 92 ms), QTc prolongation (519 ms), and ST depression. B) On day 14 of illness, we observed sinus tachycardia with occasional supraventricular premature complexes and T-wave inversion. C) On day 25, we observed widespread T-wave inversion, which persisted even after clinical recovery.

The patient was transferred to our hospital on day 10 with a diagnosis of diphtheria myocarditis. At arrival, he was afebrile, heart rate was 80 beats/min, blood pressure was 85/55 mm Hg, respiratory rate was 24 breaths/min, and oxygen saturation was 98% on room air. No pseudomembrane was visible. Cardiovascular and respiratory examinations were unremarkable. A full blood count revealed a mild leukocytosis of 17.02 k/UL and a neutrophil percentage of 70%. Renal and liver function and coagulation tests were with reference ranges, but troponin I was elevated (14,313.4 pg/mL [reference range <400 pg/mL]). An ECG showed sinus arrhythmia, incomplete right bundle branch block, and QTc prolongation (Figure 3). Cardiac point-of-care ultrasound (POCUS), which was performed early, revealed a mildly reduced EF of 57% and a cardiac output of 3.1 L/min. The result of a repeat throat swab culture was sterile. Because this patient had poor prognostic factors, including presence of a bull neck and cardiac complications, he was given DAT at a dose of 40,000 IU despite the late stage of the illness. We continued him on oral erythromycin.

**Figure 4:**
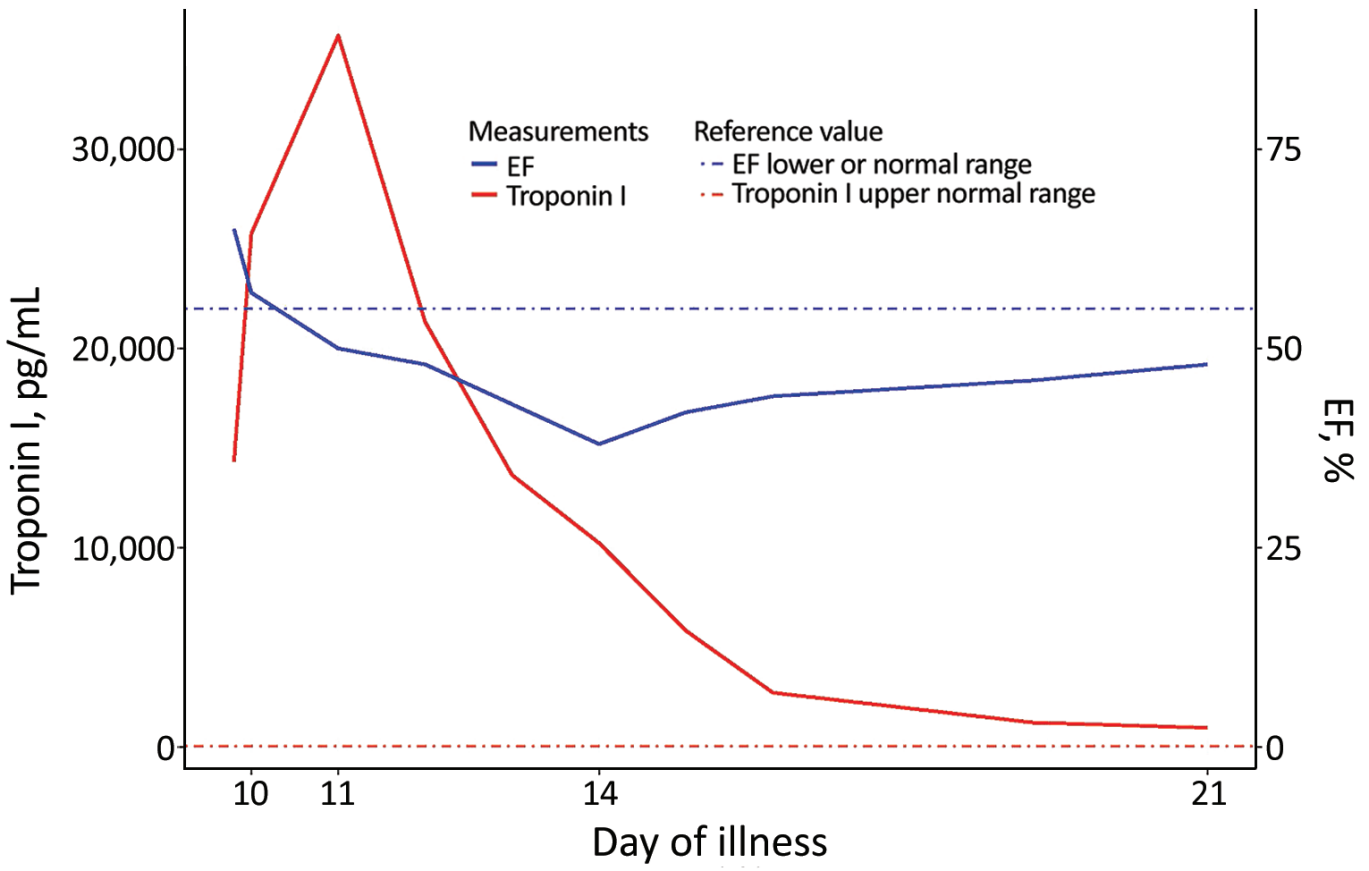
Temporal changes in troponin I levels and ejection fraction measurements during the acute phase of diphtheria myocarditis in a 7-year-old boy, Vietnam, 2020. EF, ejection fraction.

On day 11, his blood pressure reduced slightly to 80/55 mm Hg, and his heart rate was 90 beats/min. Although the ECG showed sinus rhythm with QTc prolongation (510 ms), POCUS revealed further reduction in EF to 50% and cardiac output to 2.5L/min. We immediately started an infusion of a low dose (3 μg/kg/min) of dobutamine. Troponin I had more than doubled, to 35,705.5 pg/mL (Figure 4). On day 14, the boy became more hemodynamically unstable. We performed serial POCUS to assess the extent of myocardial impairment and guide inotropic support. LV function deteriorated substantially, to an EF of 40% (Figure 4) and cardiac output of 1.54 L/min. ECG showed sinus tachycardia and T-wave inversion (Figure 4). We increased dobutamine to 8 μg/kg/min and added noradrenaline (0.1 μg/kg/min). The need for inotropic support decreased over the next 5 days, and myocardial function improved. His troponin I level normalized on day 21.

On day 39, the patient experienced neurologic symptoms, including dysphagia, dysphonia, and loss of power and sensation in his lower limbs. He subsequently had onset of respiratory distress, requiring intubation for mechanical ventilation on day 47. He gradually recovered and was weaned off the ventilator on day 62. After a week of mobilization and physiotherapy, he was discharged on day 71. At discharge, he still had mild lower limb weakness but was otherwise fully mobile. ECG results were unremarkable, and POCUS showed some residual impairment of LV function (EF of 53% and mild dilation of the left ventricle).

**Figure 5:**
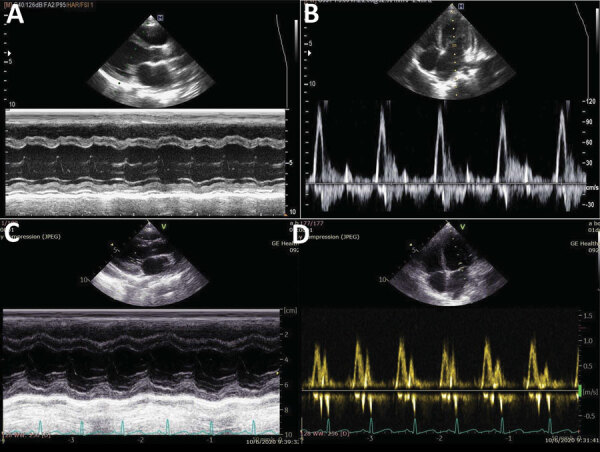
Serial echocardiographic recordings for 7-year-old boy later diagnosed with diphtheria myocarditis, Vietnam, 2020. On day 14 of illness, the M-mode left ventricular ejection fraction decreased to 40% (A) and the E/A ratio was >3.5 (B). At 2-week follow-up after discharge, both left ventricular systolic (C) and diastolic function (D) had recovered.

At a follow-up appointment 2 weeks later, the patient was well, had no cardiac symptoms, and was fully mobile. ECG results were unremarkable, and LV systolic function had improved to 60% (Figure 5), but the left ventricle remained mildly dilated at 4.3 cm.

## Diagnosis and Clinical Features of Diphtheria

*Corynebacterium* spp. are gram-positive, nonmotile rods with a club-like morphology and are aerobic or facultatively anaerobic (*13*). The infectious agents causing diphtheria are toxigenic strains of *C. diphtheriae* and, less commonly, the closely related *C. ulcerans* and *C. pseudotuberculosis*. The World Health Organization laboratory diagnostic criteria require either isolation of *C. diphtheriae* from a clinical specimen or a >4-fold rise in antibodies (*10*). The diagnosis should be confirmed by identifying 1 of the 3 species and testing for toxigenicity. The species can be identified with high sensitivity and specificity by using matrix-assisted laser desorption/ionization time-of-flight mass spectrometry. Alternatively, PCR may be used to identify the species and the presence of the *tox* gene. Toxigenicity can be confirmed with the Elek test. Diphtheria can be transmitted through respiratory droplets or direct physical contact with contagious cutaneous lesions (*3*).

Clinical symptoms typically begin 2–5 days after nasopharyngeal infection, and can include sore throat, malaise, cough, hoarse voice, painful swallowing, bloody nasal discharge, and drooling of saliva (*14*). Fever is frequently mild or absent. A gray-white membrane is characteristic, initially covering the tonsils then rapidly spreading to the uvula, soft palate, and posterior wall of the throat. In severe cases, airway obstruction can cause respiratory distress. Systemic disease occurs when diphtheria toxin disseminates in the bloodstream, leading to toxin-mediated damage to the heart, kidneys, and peripheral nerves (*11*). Notably, the heparin-binding epidermal growth factor–like growth factor, which is binding site of the diphtheria toxin, is expressed abundantly on the cell membranes of cardiomyocytes and neurons, enabling the toxic effects of diphtheria in these tissues (*13*). Neurologic disease can involve peripheral polyneuropathy and, less frequently, bulbar palsy.

Once the toxin enters the bloodstream, patients appear toxic, pale, and lethargic. Myocarditis often manifests as life-threatening arrhythmias and can be followed by neuritis and paralysis of the limbs, soft palate, and diaphragm, which can persist for weeks to months. Other known complications include otitis media, kidney injury, liver dysfunction, and thrombocytopenia (*13*,*15*). Diphtheria’s case-fatality rate ranges from 5% to 17%, but rates have improved with supportive care and DAT (*13*).

Managing diphtheria includes early use of DAT and antibiotics. Mortality rates increase daily with delays in DAT administration, from 4.2% if the disease is treated in the first 2 days to 24% by the fifth day of illness (*12*). A global shortage of DAT persists because of lower demand, resulting in former DAT manufacturers discontinuing production; the shortage is rapidly becoming a public health crisis not only in LMICs but also in high-income settings (*16*). Diphtheria can be treated effectively with several antibiotics, including penicillins, macrolides, oxacillin, clindamycin, rifampin, and tetracycline (*11*). The antibiotics of choice are erythromycin or penicillin for 14 days (*15*). However, resistance to the empirical antibiotics has been reported (*17*,*18*). Patients should be isolated for >6 days after the administration of antibiotic therapy or until 2 negative cultures are obtained.

Appropriate supportive care is also important. Tracheotomy is indicated for respiratory distress because of airway obstruction. Steroids can help reduce local inflammation but do not prevent complications (*19*). Patients should still receive primary vaccination if unvaccinated or booster vaccinations after recovery to provide full future protection (*15*).

## Cardiac Manifestations

Cardiac complications are common and well documented in diphtheria because of the affinity of the diphtheria toxin for cardiac myocytes and the cardiac conduction system (*20*). Myocarditis is caused by diphtheria toxin–induced degradation of actin filaments, leading to impaired contractile function (*21*). In patients who recover, damaged cardiomyocytes are eventually replaced with fibrotic tissue, which can lead to long-term cardiac sequelae (*13*). Cardiac complications can also occur in patients infected with nontoxigenic strains of *C. diphtheriae* (*22*,*23*).

Cardiac involvement in diphtheria is diverse but most commonly characterized by myocardial dysfunction and arrhythmias and, occasionally, pericarditis and endocarditis (*24*). The presence of a bull neck and the amount of pharyngeal pseudomembrane at admission are potential risk factors for cardiomyopathy (*25*).

Diphtheria myocarditis occurs in 10%–20% of respiratory diphtheria (*13*), although this figure is likely underestimated because cardiac screening is lacking in many endemic settings. Of note, this complication almost exclusively occurs in persons who are unvaccinated or incompletely vaccinated. Myocarditis usually manifests at the end of the second week of infection but in severe infections can manifest earlier (*26*). Diphtheria myocarditis once had a case-fatality rate of 60%–70% (*27*), but recent articles have reported a mortality rate ranging from 0% to 80% (*24*,*25*,*28*–*40*). This large discrepancy can be explained by heterogeneity of diagnostic criteria and immunization status of patients. The utility of modern diagnostic and monitoring methods, such as invasive blood pressure monitoring, continuous ECG monitoring, and point-of-care echocardiography, could also improve diagnosis and management through earlier detection of cardiac dysfunction and subclinical rhythm disturbances. Long-term sequelae in survivors of diphtheria myocarditis are rare, but sudden cardiac death can occur several months after the acute infection, suggesting persistence of a low-grade cardiomyopathy (*28*,*41*,*42*).Ventricular ectopics at admission are predictive of poor outcomes. Tachyarrhythmias and bradyarrhythmias are common and can last well into recovery (*28*). We summarized the common features of diphtheria myocarditis from case series, case reports, and observational studies conducted since 1960 (Appendix Tables 2, 3).

## Synthesized Findings of Diphtheria Myocarditis Derived from the Literature

### Laboratory Biomarkers

Laboratory markers predictive of poor outcome in diphtheria myocarditis include leukocytosis (>25 × 10^9^ leukocytes/L [reference range 3.6–11.0 leukocytes]) and elevated aspartate transaminase levels (>80 U/L [reference range 8–33 U/L]) (*24*). Marked elevation of cardiac enzymes is also associated with fulminant cardiac failure (*29*). The troponin I level likely reflects myocardial damage and the severity of the disease; however, the association between troponin I and mortality rates is still unclear (*43*). In our patient, we observed a lag between troponin I levels and EF; as troponin levels began to gradually normalize, EF continued to deteriorate for the next 3 days. Thus, even when active myocardial damage has ceased, myocardial function recovery may take more time, demonstrating the importance of using POCUS in patient monitoring.

### ECG Monitoring

ECG abnormalities in diphtheria myocarditis include atrioventricular conduction disturbances, bundle branch block, ST depression, T-wave inversion, or some combination (*25*,*30*). Prolongation of the QT or PR intervals, sinus arrhythmia, ventricular tachycardia, and supraventricular tachycardia have also been demonstrated (*25*,*30*). Complete heart block is strongly associated with severe disease and higher mortality rates (*25*,*30*). A study recording 24-hour ECGs in severe pediatric diphtheria showed high rates of supraventricular and ventricular ectopic rhythms without evidence of heart failure (*3*1). Furthermore, ventricular ectopics on admission were predictive of poor outcomes (*28*). Tachyarrhythmias and bradyarrhythmias are common and can last well into recovery (*28*). Left bundle branch block has been shown to be an independent predictor of long-term risk for death (*44*). Hence, continuous ECG monitoring, where available, should be applied to capture these changes and preempt malignant arrhythmias to improve the overall outcome in patients with severe diphtheria.

Recent advances in medical sensors and data analysis have helped with early diagnosis and close monitoring of patients with critical illness in high-income settings and, more recently, low- and middle-income settings (*45*). These devices hold promise for patients with neglected infections such as diphtheria because of their low cost, noninvasive nature, and potential for sharing findings electronically, thereby enabling remote monitoring in disease outbreak situations (*45*–*47*). An example of this approach is being evaluated for use in treatment of tetanus by applying machine learning techniques to analyze 24-hour ECG waveforms, detected by a novel low-cost wearable patch (*46*). Continuous physiologic monitoring aids in the early detection of deterioration and complications, improving the quality of care. Noninvasive and low-cost devices for continuous physiologic and ECG monitoring would thus be of huge benefit to the clinical management of diphtheria myocarditis and should be considered where available.

### Echocardiography

Echocardiography is a useful noninvasive tool to assess cardiac function. However, until now, the use of echocardiography in the diagnosis and management of diphtheria has been limited, partly because of a lack of devices and expertise in LMICs. A large knowledge gap regarding echocardiographic findings in diphtheria myocarditis exists (*43*,*48*,*49*). Patients with severe diphtheria can have no clinical evidence of heart failure despite having subtle changes in cardiac function and subclinical dysrhythmias. Abnormalities on echocardiography include both systolic and diastolic dysfunction, characterized by rapid reduction in LV EF (*24*). Other abnormalities visualized on echocardiograms are LV dilation, LV wall thickening, pericardial effusion, and mitral and tricuspid regurgitation (*24*,*48*). Serial myocardial contractility assessment using POCUS can identify early cardiac decompensation and need for inotropic support. In the case of the patient described in this article, the use of POCUS helped attending clinicians detect early deterioration in cardiac function and guide the use of inotropes and fluid resuscitation. Thus, serial cardiac POCUS together with ECG monitoring should be applied to all patients hospitalized with diphtheria because the manifestations of diphtheria myocarditis are variable (*48*). Improved availability of low-cost portable echocardiograms and increasing expertise in many intensive-care units in LMICs, in addition to wearable ECG monitors, means a comprehensive cardiac assessment is now possible and should be applied to diphtheria patients to enable timely interventions and appropriate support (*50*).

### Cardiac-Specific Treatment

Current management of diphtheria myocarditis mainly relies on supportive treatment aiming to maintain normal hemodynamic parameters. Antiarrhythmic drugs are usually reserved for sustained tachyarrhythmias. Prophylactic treatment of subclinical arrhythmias is not recommended, but further research into early administration for certain arrhythmias that are at high risk for progression or are associated with poor outcomes is warranted (*28*). Temporary pacemaker insertion can be used in patients with severe diphtheria myocarditis and bradyarrhythmias. The success of temporary pacing depends on the degree of damage to the conduction system and myocardial reserve. One report demonstrated improved survival outcomes of patients with diphtheria myocarditis and complete heart block (*32*). However, pacing did not improve outcomes in severe cases reported in Chile and Thailand (*33*,*49*).

## Discussion

The findings of our literature review are well reflected in the clinical journey of this patient, whose vaccination status was unknown. He had swollen neck and extensive pseudomembrane, followed by onset of myocarditis in the second week (day 9), despite a short period of clinical stability. He did not have risk factors for myocarditis, such as leukocytosis and elevated aspartate transaminase, and only minor cardiac rhythm disturbances were present, suggesting a good prognosis. Because DAT was only available in some health facilities in Vietnam, the patient only received DAT on day 10, when he was admitted to our hospital. Whether the use of DAT at such a late time can still alter the progression of myocarditis is unclear. In the case of this patient, myocardial function continued to deteriorate after DAT was administered. We demonstrated that the use of POCUS assisted early detection of myocardial function deterioration, whereas the ECG findings were not as informative in terms of indicating cardiac dysfunction. Moreover, cardiac troponin I levels peaked and fell despite the ongoing cardiac impairment, suggesting a time lag between myocardial cellular damage and functional performance. We therefore recommend continuing close hemodynamic monitoring until myocardial functional recovery can be visualized on echocardiographic examination. Continuous monitoring using the POCUS and low-cost wearables coupled with intelligent technology is now under investigation in our hospital for treatment of other neglected diseases in Vietnam (http://vital.oucru.org). Positive results should encourage the extrapolation of this approach to other LMIC settings.

In conclusion, we report a severe case of diphtheria myocarditis, monitored using serial point-of-care echocardiography, enabling the timely management of the cardiac complications that ensued, which included heart failure and rhythm abnormalities. Serial point-of-care echocardiography, where available, together with serial ECGs and standard-of-care clinical monitoring, should be used in the treatment of all hospitalized patients with diphtheria myocarditis to enable timely intervention to mitigate severe and life-threatening consequences. Noninvasive wearable technology might be of benefit for detecting early signs of deterioration in diphtheria myocarditis. Outbreaks of diphtheria may become more frequent in the future because of the ongoing COVID-19 pandemic and related health service disruption but also because of increased vaccine hesitancy in many countries. The increasing availability of portable ultrasound and low-cost monitoring devices in Vietnam and other LMICs should now mean these methods can be used as standard of care in managing diphtheria myocarditis. Access to supplies of DAT, particularly in vulnerable settings, needs to be prioritized, and awareness of diphtheria, including its potential complications and management, should be raised for frontline health workers globally. Finally, full diphtheria vaccination provides the best protection against all complications of diphtheria.

Appendix Additional information about novel clinical monitoring approaches for reemergence of diphtheria myocarditis, Vietnam.
