# Supplementary material for: Novel Clinical Monitoring Approaches for Reemergence of Diphtheria Myocarditis, Vietnam
Source: Emerg Infect Dis. 2022 Feb;28(2):282–90. doi: 10.3201/eid2802.210555 (PMC8798685; doi:10.3201/eid2802.210555)
Supplement: Appendix — Additional information about novel clinical monitoring approaches for reemergence of diphtheria myocarditis, Vietnam. [file 21-0555-Techapp-s1.pdf]

Article DOI: https://doi.org/10.3201/eid2802.210555

Novel Clinical Monitoring Approaches for Reemergence of Diphtheria Myocarditis, Vietnam

Appendix

Search Methods

PubMed was searched for published articles from 1 January, 1960, to 14 August 2020 by using the following terms: (“Diphtheria”[Mesh).OR “Diphtheria Toxin”[Mesh).OR “Corynebacterium diphtheriae” OR “C diphtheriae”) AND (“myocarditis” OR “Heart Block”[Mesh).OR “Arrhythmias, Cardiac”[Mesh).OR “Cardiac Conduction System Disease”[Mesh).OR “Cardiomyopathies”[Mesh).OR “Ventricular Dysfunction”[Mesh).OR “Heart Valve Diseases”[Mesh).OR “Echocardiography”[Mesh]). In addition, references from relevant articles were also searched. Articles relevant to the subject matter of the review and published in English, French, and German were included.

Appendix Table 1. List of review papers and citations

Number	Citation
1	James TN, Reynolds EWJ. Pathology of the cardiac conduction system in a case of diphtheria associated with atrial arrhythmias and heart block. Circulation 1963; 28:263–267.
2	Class RN, Rivera-Gallegos BT, Sánz-Malaga G. Diphtheritic myocarditis simulating myocardial infarction. Am J Cardiol 1965; 16:580–584.
3	Favara BE, Franciosi RA. Diphtherial myocardiopathy. Am J Cardiol 1972; 30:423–426.
4	Matisonn RE, Mitha AS, Chesler E. Successful electrical pacing for complete heart block complicating diphtheritic myocarditis. Br Heart J 1976; 38:423–426.
5	Groundstroem KW, Molnar G, Lumio J. Echocardiographic follow-up of diphtheric myocarditis. Cardiology 1996; 87:79–81.
6	Perles Z, Nir A, Cohen E, Bashary A, Engelhard D. Atrioventricular block in a toxic child: do not forget diphtheria. Pediatr Cardiol 2000; 21:282–283.
7	Lakkireddy DR, Kondur AK, Chediak EJ, Nair CK, Khan IA. Cardiac troponin I release in non-ischemic reversible myocardial injury from acute diphtheric myocarditis. Int. J. Cardiol. 2005; 98:351–354.
8	Varghese MJ, Ramakrishnan S, Kothari SS, Parashar A, Juneja R, Saxena A. Complete heart block due to diphtheritic myocarditis in the present era. Ann Pediatr Cardiol 2013; 6:34–38. Available at: /pmc/articles/PMC3634244/?report = abstract.
9	Washington CH, na ayuthaya S, Makonkawkeyoon K, Oberdorfer P. A 9-y-old boy with severe diphtherial infection and cardiac complications. BMJ Case Rep 2014; 2014.
10	Skogmar S, Tham J. Severe diphtheria with neurologic and myocardial involvement in a Swedish patient: a case report. BMC Infect Dis 2018; 18:359.
11	Van Damme K, Peeters N, Jorens PG, et al. Fatal diphtheria myocarditis in a 3-y-old girl-related to late availability and administration of antitoxin? Paediatr Int Child Health 2018; 38:285–289.
12	Claman HN. Progressive myocardial damage following recovery from diphtheria. A case showing development of complete heart block. Am J Cardiol 1962; 9:790–794.
13	Moffat RC. Diphtheritic heart block. A case report demonstrating the progressive development of complete heart block and treatment with a pacemaker. Angiology 1972; 23:609–616.

Number	Citation
14	Gerlis LM, Anderson RH, Becker AE. Complete heart block as a consequence of atriondal discontinuity. Br Heart J 1975; 37:345–356.
15	Choremis C, Leonidas J. Serum transaminases in diphtheritic myocarditis. Their relation to electrocardiographic findings. Acta Paediatr 1962; 51:293–302.
16	Ledbetter MK, Cannon AB 2nd, Costa AF. The electrocardiogram in diphtheritic myocarditis H. Am Heart J 1964; 68:599–611.
17	Tahernia AC. Electrocardiographic abnormalities and serum transaminase evels in diphtheritic myocarditis. J Pediatr 1969; 75:1008–1014.
18	Wahab AS, Ismangun, Sastroamidjojo P. A selective study of clinical diphtheric myocarditis. Paediatr Indones 1973; 13:24–30.
19	Kazemi B, Tahernia AC, Zandian K. Motor nerve conduction in diphtheria and diphtheritic myocarditis. Arch Neurol 1973; 29:104–106.
20	Ismail D, Utomo, Suharjanto. Serum transaminase in diphtheritic myocarditis. Paediatr Indones 1976; 16:89–93.
21	Prasodo AM, Narendra M, Joernil A, Wahjoenarso, Kaspan F. Congestive heart failure in diphtheric myocarditis. Paediatr Indones 1976; 16:71–80.
22	Stockins BA, Lanas FT, Saavedra JG, Opazo JA. Prognosis in patients with diphtheric myocarditis and bradyarrhythmias: assessment of results of ventricular pacing. Br Heart J 1994; 72:190–191.
23	Bethell DB, Dung NM, Loan HT, et al. Prognostic value of electrocardiographic monitoring of patients with severe diphtheria. Clin Infect Dis 1995; 20:1259–1265.
24	Loukoushkina EF, Bobko P V, Kolbasova E V, et al. The clinical picture and diagnosis of diphtheritic carditis in children. Eur J Pediatr 1998; 157:528–533.
25	Havaldar P V, Sankpal MN, Doddannavar RP. Diphtheritic myocarditis: Clinical and laboratory parameters of prognosis and fatal outcome. Ann Trop Paediatr 2000; 20:209–215. Available at: https://pubmed.ncbi.nlm.nih.gov/11064774/.
26	Kneen R, Dung NM, Solomon T, et al. Clinical features and predictors of diphtheritic cardiomyopathy in Vietnamese children. Clin Infect Dis 2004; 39:1591–1598. Available at: https://academic.oup.com/cid/article-lookup/doi/10.1086/425305.
27	Kole A, Roy R, Kar S. Cardiac involvement in diphtheria: Study from a tertiary referral infectious disease hospital YR - 2012/7/1. Ann Trop Med Public Heal 2012; 5:302–306.
28	Samdani S, Jain A, Meena V, Meena CB. Cardiac complications in diphtheria and predictors of outcomes. Int J Pediatr Otorhinolaryngol 2018; 104:76–78.
29	Thisyakorn U, Wongvanich J, Kumpeng V. Failure of corticosteroid therapy to prevent diphtheritic myocarditis or neuritis. Pediatr Infect Dis 1984; 3:126–128.
30	Ramos AC, Elias PR, Barrucand L, Da Silva JA. The protective effect of carnitine in human diphtheric myocarditis. Pediatr Res 1984; 18:815–819.
31	Dung NM, Kneen R, Kiem N, et al. Treatment of severe diphtheritic myocarditis by temporary insertion of a cardiac pacemaker. Clin Infect Dis 2002; 35:1425–1429
32	Celik T, Selimov N, Vekilova A, et al. Prognostic significance of electrocardiographic abnormalities in diphtheritic myocarditis after hospital discharge: a long-term follow-up study. Ann Noninvasive Electrocardiol 2006; 11:28–33

Appendix Table 2. Summary of reported cases with diphtheria myocarditis since 1960

Reference	Year	Country of disease origin	Case reported	Immunization status	Microbiology	Cardiac features	Treatment	Outcome
Acute phase								
James and Reynolds	1963	United States	1 (2.5/F)	NA	*C. diphtheriae mitis*	Progressive major conduction disturbances; atrial flutter, atrial fibrillation. ECG: right bundle branch block, 2:1 AV block. Histopathology: generalized myocarditis with vacuolization degeneration of fibers in conduction tissues	DAT, Penicillin, Tracheotomy, Digoxin	Died
Class et al.	1965	Peru	1 (13/M)	NA	*C. diphtheriae*	No clinical symptoms of myocarditis ECG: right bundle branch block, ST elevation, and QS complexes in V1–3 lead. AST: 50 IU/L	DAT, Penicillin	Survived
Favara and Franciosi	1972	United States	1 (7/M)	No	Toxigenic *C. diphtheriae*	Progressive cardiac arrhythmias. ECG: 2:1 AV block	No DAT, Penicillin, Digoxin	Died
Matisonn et al.	1976	South Africa	1 (8/M)	NA	Negative for *C. diphtheriae*	Bradycardia and hypotension ECG: 2nd degree AV block, complete LBBB. Left anterior hemiblock and anterior myocardial damage with widening QRS complexes	DAT, Penicillin, Tracheostomy, Mechanical ventilation, Isoprenaline Temporary pacemaker	Survived
Groundstroem et al.	1996	Russia	1 (43/M)	NA	*C. diphtheriae*	No clinical signs of myocarditis. ECG: QT prolongation, ST elevation and T wave inversion. Echo: Hypokinesia LV anterior wall with normal contraction, new diastolic dysfunction. Normal cardiac function at 5-mo follow-up. CK: 1281 U/L	No DAT, Erythromycin	Survived
Perles et al.	2000	Israel	1 (4.5/F)	No	*C. diphtheriae*	Progressive severe arrhythmias: severe bradycardia, ventricular tachycardia, leading to cardiogenic shock. ECG: Complete AV block Normal echocardiogram at admission. CK: 94 U/L	No DAT, Erythromycin, Penicillin, Gentamicin, Lidocaine, Temporary pacemaker, Vasopressors, Platelet transfusion	Died
Lakkireddy et al.	2005	United States	1 (20/F)	NA	Negative for *C. diphtheriae* but Positive PCR for diphtheria toxin	Tachycardia with summation gallop ECG: sinus tachycardia with non-specific ST-T wave changes. Echo: Impaired LV systolic function with LVEF of 10%. Normal cardiac function at 6-mo follow-up. Troponin I: 48.5 ng/ml	DAT, Ampicillin, Tracheostomy, Digoxin Furosemide, ACE inhibitor	Survived
Varghese et al.	2013	India	6 cases	3 no, 2 partial, 1 unknown	2/6 with positive for *C. diphtheriae*	Bradycardia and cardiogenic shock (3/6). ECG: Complete heart block, left bundle branch block. Echo: Global hypokinesia, LV systolic dysfunction (EF: 15%–50%), mitral regurgitation and tricuspid regurgitation, pericardial effusion	DAT (3/6), Erythromycin, Penicillin, Inotropes, Temporary pacemaker	3 died (all unimmunized), 3 survived (1 with persistent CHB)

Reference	Year	Country of disease origin	Case reported	Immunization status	Microbiology	Cardiac features	Treatment	Outcome
Washington et al.	2014	Thailand	1 (9/M)	No	*C. diphtheriae;* Positive Elek test	Progressive cardiac arrhythmias. ECG abnormalities: ectopic atrial rhythm, incomplete RBBB, progressing to 3rd degree AV block, LBBB. Echo: Severe myocardial dysfunction function (EF = 8%), tricuspid regurgitation, pericardial effusion. CK-MB: 7.6 ng/ml, Troponin T = 74 ng/ml, Pro-BNP: 1236 ng/L	DAT, Penicillin, Cefotaxime, Milrinone Temporary pacemaker, Methylprednisolone, IVIG	Died
Skogmar et al.	2018	Sri Lanka	1 (55/M)	Unknown	*C. diphtheriae non-gravis*	No clinical symptoms of myocarditis ECG: T-wave inversion and ventricular tachycardia	No DAT, Cefotaxime, Erythromycin, Tracheostomy, Mechanical ventilation	Survived
Van Damme et al.	2018	Belgium	1 (3/F)	Partial	*C. diphtheriae*	Progressive conduction disturbances. ECG: 3rd degree AV block. Echo: maintained LV systolic function (EF = 60%), mitral regurgitation 2/4, tricuspid regurgitation 3/4, hyper-echogenic myocardium	DAT, Amoxicillin, Clindamycin, Isoprenaline, Inotropes, Temporary pacemaker	Died
Chronic phase								
Claman	1962	Germany	1 (36/F)	NA	NA	Acute diphtheria at age 24 y, with myocarditis and bundle branch block noted on ECG. She developed progressive congestive heart failure over 7 y of follow-up with complete AV block in ECG	Low sodium diet, Diuretics, Digoxin	Survived
Moffat	1972	Germany	1 (46/M)	NA	NA	Pharyngeal diphtheria with cardiac and neurologic complications at the age of 20 y. Bradycardia on ECG during acute illness. 25 y after the acute illness, he developed syncopal episodes and a progressive complete heart block requiring the insertion of permanent cardiac pacemaker.	Cardiac pacemaker, Lidocaine	Survived
Gerlis et al.	1975	United Kingdom	1 (39/F)	NA	NA	Acute diphtheria at the age of 2 y with myocarditis and heart block on ECG as well as neurologic complications. At the age of 15 y, she developed signs of heart failure and complete heart block on ECG. Sudden cardiac death at the age of 32 y.	Periodic follow-up, No treatment	Died

ACE inhibitor: angiotensin-converting-enzymes inhibitor; AST: aspartate aminotransferase; CHB: complete heart block; CK: creatinine kinase; CK-MB: creatinine kinase – myocardial band; DAT: diphtheria antitoxin; ECG: Electrocardiogram, F: Female; IVIG: intravenous immunoglobulin; LBBB: left bundle branch block; LVEF: left ventricular ejection fraction; M: Male; NA: not applicable; Pro-BNP: pro-B type natriuretic peptide; RBBB: right bundle branch block.

Appendix Table 3. Summary of clinical studies of diphtheria myocarditis since 1960

Reference	Year	Country of disease origin	Diphtheria myocarditis cases*	Mortality rate % (deaths/ total)	Main findings	Comments
Choremis et al.	1962	Greece	19 (acute phase) 10 (chronic)	31.5 (6/19)	An increase in AST and ALT levels occurred along with the onset of ECG changes in diphtheria myocarditis. AST levels were higher and increased more consistently than ALT levels	Study of 49 children with diphtheria in 1961 admitted to a local hospital aiming to find prognostic factors
Ledbetter et al.	1964	United States	47	31.9 (15/47)	Patients with prolonged PR interval and primary T-wave changes had good prognosis, those with intraventricular block and complete bundle branch block and complete A-V block had poor prognosis. Pathological findings included -Extensive myocardial disease, hemorrhage and interstitial edema of myocardium in patients who experienced major conduction disturbances and died early.	Study of 229 children with diphtheria between 1954–1960 admitted to a local hospital aiming to find prognostic factors and relate ECG changes with clinical and pathologic findings.
Tahernia	1969	Iran	21	47.6 (10/21)	AST and ALT levels had good correlation with the severity of ECG abnormalities in patients with diphtheria myocarditis, and the degree of ECG abnormalities correlated well with the clinical status. Serial transaminase measurements and ECGs could be used as diagnostic and prognostic factors	Study of 46 children with diphtheria between 1964–1967 admitted to a local hospital aiming to find prognostic factors.
Wahab et al.	1973	Indonesia	47	0 (0/47)	The selected cases, had higher socio-economic status and had subclinical myocarditis with very mild ECG abnormalities changes, such as sinus tachycardia, PR prolongation, ST depression with or without T wave changes.	Study of 92 children with diphtheria during 1970–1971 admitted to a local hospital aiming to explore the prognosis of patients with diphtheria myocarditis.
Kazemi et al.	1973	Iran	11	Not given	All diphtheria patients had delayed conduction velocity of median, ulnar, and common peroneal nerves. More profound delays in conduction velocities were seen in those with cardiac involvement.	Study of 30 children with diphtheria between 1969–1971 admitted to 2 hospitals aiming to explore the peripheral nerve conduction in diphtheria
Ismail et al.	1976	Indonesia	34	17.6 (6/34)	Good correlation between AST levels and the severity of ECG abnormalities in diphtheria patients.	Study of 91 children with diphtheria between 1973–1974 admitted to a single hospital aiming to find prognostic factors.
Prasodo et al.	1976	Indonesia	331	27.4 (92/331)	Congestive heart failure, characterized by hepatic enlargement +- epigastric pain, and dyspnea, noted in 50% of patients with severe ECG changes including AV block, RBBB and LBBB and only in 5% of those with ST depression or T-wave changes. Digitalis treatment did not improve fatal outcome in patients with congestive heart failure.	Study of 331 diphtheria patients with abnormal ECGs from 1969–1974 admitted to a single hospital, aiming to evaluate incidence, characteristics and outcome of heart failure in diphtheria myocarditis
Stockins et al.	1994	Chile	46	45.8 (11/24 bradyarrhythmias)	Intraventricular conduction, and 3rd degree AV block were markers of severe myocardial damage and patients with carried a poor prognosis. Those with sinus bradycardia and AV dissociation had a better prognosis. Ventricular pacing did not improve survival in cases with 3rd degree AV block.	Study of 167 diphtheria patients between 1976–1986 admitted to a hospital to evaluate the prognosis of brady arrhythmias and assess the results of ventricular pacing in those with 3rd degree AV block
Bethell et al.	1995	Vietnam	15	33.3 (5/15)	All patients with severe diphtheria had subclinical ECG abnormalities, particularly high rates of supraventricular and ventricular ectopy, on 24-h recordings during hospitalization and were observed until discharge. The number of ventricular ectopics at the time of presentation was associated with fatal outcome (>2 VE at admission predicted fatal outcome with 100% sensitivity and 100% specificity)	Study of 15 patients with severe diphtheria between 1991–1992 admitted to intensive care in 1 hospital
Loukoushkin a et al.	1998	Russia	64	14.1 (9/64)	Rapid development of cardiac failure with a variety of conduction disturbances seen in ECG. Echocardiograms	Study of 122 children with diphtheria between1992–1996, admitted to one

Reference	Year	Country of disease origin	Diphtheria myocarditis cases*	Mortality rate % (deaths/ total)	Main findings	Comments
					showed LV dilatation, depressed LV function. Hypermyoglobinaemia (>2000 ng/ml) and an increased LDH1/LDH2 (>1) ratio were prognostic markers for the development of myocarditis and poor prognosis.	hospital, aiming to describe diphtheria myocarditis
Havaldar et al.	2000	India	16	50 (8/16)	Fatal outcome was associated with extension of membrane formation to two or more sites, total leukocyte counts of >25,000 cells/mm3 and AST levels of >80 IU/L	Study of 97 children with diphtheria between1989–1995 admitted to a hospital aiming to investigate predictors of mortality.
Kneen et al.	2004	Vietnam	32	37.5 (12/32)	13 patients had myocarditis at admission, and 19 developed myocardidits during the admission. The combination of pseudomembrane score of >2 and bull neck could predict the development of diphtheria cardiomyopathy. 24-h ECG recordings on admission can improve the prediction of diphtheria cardiomyopathy. Fatal outcome was best predicted by the combination of myocarditis on admission, a pseudomembrance score of >2, elevated AST level (>47 IU/L).	Study of 154 children with diphtheria between 1995–1996 admitted to a hospital investigating diagnostic and prognostic factors
Kole et al.	2012	India	70	2.6 (2/70)	ECG changes were often subclinical and included T-wave inversion in sinus tachycardia, ST segment depression, AV block, right bundle branch block, multiple atrial ectopics, and sinus bradycardia. AST levels were elevated consistently in patients with features of cardiac failure.	Study of 100 diphtheria cases between 2010–2011 admitted to a hospital aiming to describe diphtheria myocarditis
Samdani et al.	2018	India	60	25 (15/60)	ECG changes included sinus tachycardia, T-wave inversion, ST segment depression, RBBB and multiple atrial ectopics. CK-MB level more than >4.3 ng/ml, myoglobulin levels of more than 107 ng/ml, and Troponin T >0.4ng/ml were associated mortality	Study of 158 diphtheria patients admitted to a hospital over 3 y aiming to find predictors of outcomes
Thisyakorn et al.	1984	Thailand	17	Not given	31 patients were randomly assigned to take oral prednisolone (2mg/kg/day) for 2 weeks, 35 did not receive any intervention. No significant difference in frequency of ECG changes and neuritis between the two groups. Steroid therapy had no beneficial effect in the prevention or treatment of diphtheria myocarditis and neuritis	A randomized trial of 66 patients with confirmed diphtheria between1980–1982 investigating the efficacy of corticosteroid therapy on preventing myocarditis and neuritis.
Ramos et al.	1984	Brazil	111	Not given	73 were treated with carnitine supplement (100mg/kg/day) for 4 d, 59 received standard of care. Patients given Carnitine therapy had a decreased incidence of heart failure (p = 0.0475), of pacemaker implants (p = 0.0256), and mortality (p = 0.013).	A randomized trial of 132 diphtheria patients over 1 y, admitted to a hospital investigating the effect of carnitine therapy in diphtheria myocarditis
Dung et al.	2002	Vietnam	32	78.1 (25/32)	Insertion of temporary cardiac pacemaker was associated with 74% reduction in mortality of children and adolescents with diphtheria myocarditis and severe conduction defects. The need for inotropic support after pacemaker insertion was a poor prognostic sign. Echocardiographic abnormalities were dyskinesia of the intraventricular septum, dilated left ventricle, reduced ejection fraction, reduced cardiac index.	Study of 32 children and adolescents with diphtheria myocarditis and severe conduction abnormalities admitted to PICU treated with pacemaker insertion between 1992–1995 to investigate outcomes in this population
Celik et al.	2006	Turkey	32	26-mo mortality 37.5 (12/32)	32 patients were followed up for an average of 16.3 mo (10.3 – 26.8) after hospital discharge. Those with LBBB and T-wave inversion at hospital discharge had lower survival rates that	Long term follow-up study of 32 patients with diphtheria myocarditis between 1991–1996 investigating long-term cardiac

Reference	Year	Country of disease origin	Diphtheria myocarditis cases*	Mortality rate % (deaths/ total)	Main findings	Comments
					those without ECG changes. Left bundle branch block was an independent predictor of long-term mortality.	mortality and prognostic value of ECG after hospital discharge

ALT: alanine aminotransferase; AST: aspartate aminotransferase; AV: atrioventricular; CK-MB: creatinine kinase-myocardial band; ECG: electrocardiogram; LBBB: left bundle branch block; LDH: lactate dehydrogenase; RBBB: right bundle branch block. *The diagnostic criteria of diphtheria myocarditis are varying among the included studies.
